# Effects of the continuous rate infusion of propofol with or without fentanyl on ruminal motility in calves

**DOI:** 10.29374/2527-2179.bjvm010326

**Published:** 2026-10-02

**Authors:** Guilherme Lopes da Silva, Maurício Deschk, Thomas Alexander Trein, Marcelo Augusto de Araújo, Bruna de Moraes Martins Games, Celso Antônio Rodrigues, Paulo Sérgio Patto dos Santos

**Affiliations:** 1 Faculdade de Medicina Veterinária, Universidade Estadual Paulista (UNESP), Araçatuba, São Paulo, Brazil; 2 Faculdade de Medicina Veterinária e Zootecnia, Universidade Estadual Paulista (UNESP), Botucatu, São Paulo, Brazil; 3 Faculdade de Medicina Veterinária e Zootecnia, Universidade Federal de Mato Grosso do Sul (UFMS), Campo Grande, Mato Grosso do Sul, Brazil

**Keywords:** calves, rumen, anesthesia, analgesia, opioid, bezerros, rúmen, anestesia, analgesia, opioide

## Abstract

This study aimed to evaluate ruminal motility before, during, and after propofol anesthesia with or without fentanyl in Holstein calves by investigating effects of these anesthetic protocols on rumen function and cardiorespiratory variables. This study employed a randomized crossover design with a 1-week washout period between treatments and included eight calves aged 6-12 months and weighing 100-180 kg. Animals received either propofol alone (propofol group [PG]) or propofol with fentanyl (propofol-fentanyl group [FG]), alternating the order of treatments between groups. Anesthesia was induced with propofol (5.0 mg/kg IV) in the PG and with propofol (4.0 mg/kg IV) and fentanyl (0.001 mg/kg IV) in the FG. Next, anesthesia was maintained for 60 min by the continuous infusion of propofol (0.6 mg/kg/min IV) alone in the PG or combined with fentanyl (0.001 mg/kg/h IV) in the FG. Cardiorespiratory variables were recorded at baseline and every 15 min during anesthesia. Ruminal motility was assessed before fasting, after fasting, during anesthesia, and throughout recovery until contractions returned to baseline values. We noted that the median (minimum/maximum) ruminal normalization time did not differ significantly between the PG (35 [10/50] min) and FG (45 [20/120] min) (P = 0.19). Food withholding and handling-related stress reduced ruminal motility in both groups. Both protocols completely suppressed ruminal contractions during anesthesia, with contractions beginning to return approximately 10 min after the end of infusion in the PG and 20 min in the FG. Furthermore, propofol caused hypoventilation and respiratory acidosis while maintaining hemodynamic variables within clinically acceptable limits. In summary, fentanyl addition was not associated with a statistically significant prolongation of ruminal-motility recovery, although the wider range of the ruminal normalization time observed in the FG suggests individual variability, warranting further investigation.

## Resumo

O objetivo do estudo foi avaliar a motilidade ruminal antes, durante e após a anestesia com propofol, associado ou não ao fentanil, em bezerros da raça Holandesa. Foram utilizados oito bezerros com idade entre seis e 12 meses e peso entre 100 e 180 kg, submetidos a um delineamento crossover randomizado, com período de *washout* de uma semana entre os tratamentos. Os animais receberam propofol isoladamente (PG) ou propofol associado ao fentanil (FG), alternando a ordem dos tratamentos entre os grupos. A anestesia foi induzida com propofol (5,0 mg/kg IV) no PG e com propofol (4,0 mg/kg IV) associado ao fentanil (0,001 mg/kg IV) no FG. Em ambos os grupos, os animais foram intubados e posicionados em decúbito lateral direito, respirando espontaneamente ar ambiente. A manutenção anestésica foi realizada por meio de infusão contínua de propofol (0,6 mg/kg/min IV), isoladamente ou associado ao fentanil (0,001 mg/kg/h IV), durante 60 min. As variáveis cardiorrespiratórias foram registradas no momento basal e a cada 15 min durante a anestesia. A motilidade ruminal foi avaliada antes do jejum, após o jejum, durante a anestesia e ao longo da recuperação anestésica até o retorno aos valores basais. O tempo mediano (mínimo/máximo) de normalização ruminal não diferiu significativamente entre o PG [35 (10/50) min] e o FG [45 (20/120) min] (P = 0,19). O jejum alimentar e o estresse relacionado ao manejo reduziram a motilidade ruminal em ambos os grupos. Ambos os protocolos suprimiram completamente as contrações ruminais durante a anestesia, com início do retorno das contrações aproximadamente 10 min após o término da infusão no PG e 20 min no FG. O propofol causou hipoventilação e acidose respiratória, mantendo as variáveis hemodinâmicas dentro de limites clinicamente aceitáveis. A adição de fentanil não esteve associada a um prolongamento estatisticamente significativo da RMT, embora a maior amplitude do tempo de normalização observada no FG sugira variabilidade individual que requer investigação adicional.

## Introduction

Total intravenous anesthesia (TIVA) is widely used in ruminants owing to its practicality under field conditions and its ability to provide adequate anesthetic depth through different drug combinations administered using continuous rate infusion (Picavet et al., 2004; Santos et al., 2010). In calves, TIVA protocols have demonstrated satisfactory cardiorespiratory stability and represent a viable alternative to inhalational anesthesia, particularly in situations wherein structural limitations preclude the use of more complex anesthetic equipment (Sato et al., 2024). In addition, increasing concerns regarding analgesia, animal welfare, and intraoperative stability in cattle has encouraged the development of safer, more effective, and balanced anesthetic protocols for clinical use in ruminants (Dal Más et al., 2022; Sato et al., 2025).

Among intravenous anesthetics used in veterinary medicine, propofol is characterized by rapid anesthetic induction, a short duration of action, and relatively rapid recovery; these features favor its use in continuous rate infusion (Carareto et al., 2007; Duke et al., 1997). In ruminants, anesthetic induction with propofol is considered smooth, and the slow administration of the drug may reduce the occurrence of apnea (Tranquilli et al., 2007). However, studies in calves have demonstrated that continuous propofol infusion may promote hypoventilation, increased end-tidal carbon dioxide concentrations, and respiratory acidosis, particularly in animals maintained under spontaneous ventilation (Deschk et al., 2017).

Fentanyl is a μ-opioid agonist that is widely used in balanced anesthesia and TIVA protocols owing to its potent analgesic effects and its ability to reduce the requirement for higher doses of hypnotic anesthetic agents. In cattle, the recommended infusion rate is 0.001-0.002 mg/kg/h (Tranquilli et al., 2007). Recent studies in calves have demonstrated that the combination of propofol and fentanyl provides adequate anesthetic maintenance, although it may intensify respiratory depression and may prolong anesthetic recovery (Deschk et al., 2017). Furthermore, marked individual variability in plasma concentrations of fentanyl and its metabolite norfentanyl has been described in Holstein calves, suggesting pharmacokinetic differences that are capable of influencing clinical effects observed during anesthesia (Smith et al., 2018).

In addition to their cardiorespiratory effects, intravenous anesthetics and opioids may directly interfere with gastrointestinal motility in ruminants. Specifically, μ-opioid agonists are capable of inhibiting cyclic reticuloruminal contractions and altering digestive dynamics in cattle (Dal Más et al., 2022). Reticuloruminal motility plays a fundamental role in digesta movement, the absorption of ruminal fermentation products, and the elimination of gases produced within the rumen, which are essential for the maintenance of normal rumination and eructation (Constable et al., 1990). Alterations in this motility may result in ruminal hypomotility or atony, predisposing animals to ruminal distension, impaired eructation, and important digestive disturbances (Attia et al., 2021; Costa et al., 2022).

In addition to their cardiorespiratory effects, intravenous anesthetics and opioids may interfere with gastrointestinal motility, and μ-opioid agonists can inhibit cyclic reticuloruminal contractions in cattle (Dal Más et al., 2022). Reticuloruminal activity is essential for mixing ruminal contents, fermentation, fermentation-product absorption, eructation, and digesta progression through the forestomachs (Braun, 2016; Constable et al., 1990; Radostits et al., 2007). This activity is influenced by receptors that monitor reticuloruminal-wall distension: moderate distension stimulates motor activity, whereas excessive distension activates inhibitory pathways that may progressively reduce contraction strength and frequency and ultimately result in ruminal atony (Constable et al., 2017). Central nervous system depression, stress, pain, and vagal dysfunction may also contribute to ruminal hypomotility (Attia et al., 2021; Feitosa, 2014). The persistent impairment of motility may lead to ruminal distension, impaired eructation, gas and ingesta accumulation, and clinically important digestive disturbances (Costa et al., 2022; Soares et al., 2022). Therefore, the evaluation of ruminal contractions is clinically relevant in cattle undergoing general anesthesia.

Although recent studies have evaluated hemodynamic and respiratory variables and indices related to anesthetic depth during TIVA protocols in calves, information regarding effects of the combination of propofol and fentanyl on ruminal motility during the anesthetic and recovery periods remains limited (Deschk et al., 2017). Therefore, understanding effects of these anesthetic protocols on ruminal motor activity may provide relevant information for the safer use of intravenous anesthesia in cattle, particularly during procedures performed under field conditions.

The present study aimed to evaluate effects of the continuous rate infusion of propofol with or without fentanyl on ruminal motility in Holstein calves maintained under spontaneous ventilation. We hypothesized that both anesthetic protocols would reduce ruminal motility, with a greater effect observed in calves receiving fentanyl.

## Material and methods

Eight male Holstein calves, aged 6-12 months and weighing 100-180 kg, were included in the study. Animals were considered to be clinically healthy based on physical examination, including the assessment of heart rate (HR), rectal temperature, and visible mucous membrane color, in addition to complete blood-count analysis. Animals were randomly distributed: that is, four calves initially participated in the propofol group (PG) followed by the propofol-fentanyl group (FG), whereas the remaining four animals underwent the protocols in the reverse order. All animals underwent both anesthetic protocols with a 1-week interval between procedures (Figure 1). Calves were maintained in *Brachiaria* spp. paddocks and were supplemented with corn silage and commercial concentrate containing a minimum crude protein content of 160 g/kg.

**Figure 1:**
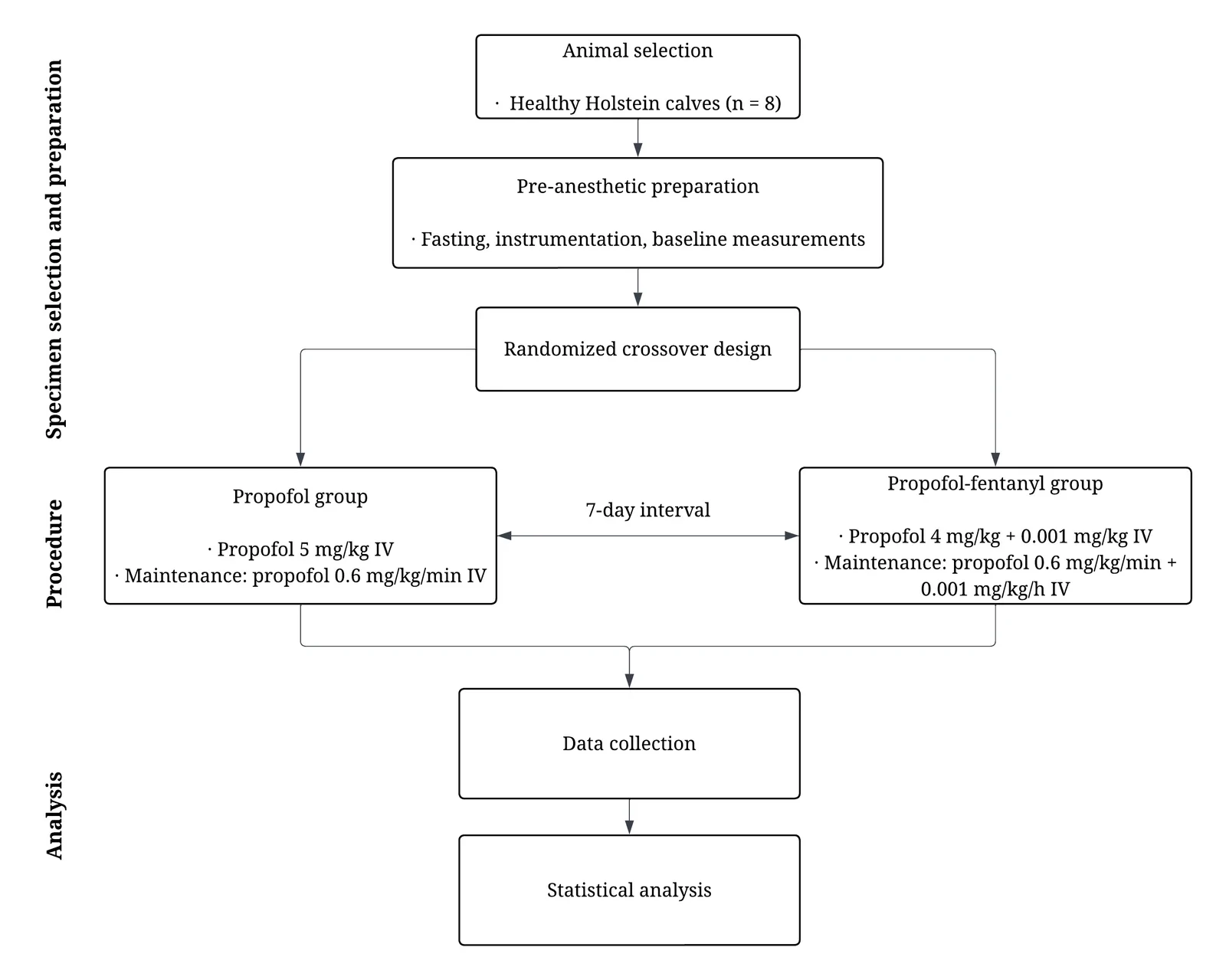
Experimental design of the randomized crossover study evaluating the effects of propofol and propofol-fentanyl anesthesia in Holstein calves.

Note. IV = intravenous.

After selection and weighing, animals were restricted from food and water for 24 and 12 hours, respectively. Subsequently, calves were physically restrained for trichotomy of the right and left jugular regions and left auricular region for catheter placement. Animals were considered healthy and classified as ASA 1 (American Society of Anesthesiologists). A 22G catheter was inserted into the left auricular artery for the measurement of systolic (SAP), diastolic (DAP), and mean blood pressures (MAP). Additional 16G catheters were placed in the right jugular vein and left cephalic vein for the intravenous administration of anesthetic drugs used in the experimental protocol, with propofol infused through the jugular vein and fentanyl infused through the cephalic vein. An introducer catheter was placed in the left jugular vein for maintenance fluid therapy using lactated Ringer’s solution administered at a rate of 5 mL/kg/h.

After preparation and instrumentation, animals were allowed to rest for 10 minutes to minimize the effects of handling-related stress on baseline values of studied variables. Thereafter, baseline measurements (BM) were recorded.

In the PG, anesthesia was induced with propofol (5 mg/kg) administered intravenously (IV). In the FG, animals received propofol (4 mg/kg) with fentanyl (0.001 mg/kg IV), both administered over a 2-min period. Immediately post-induction, animals were orotracheally intubated with appropriately sized endotracheal tubes and positioned in right lateral recumbency, where they remained under spontaneous ventilation in room air (FiO_2_ = 0.21).

Anesthetic maintenance was subsequently achieved by the continuous rate infusion of propofol at 0.6 mg/kg/min IV, with or without fentanyl at 0.001 mg/kg/h depending on the group, for 60 min using an infusion pump (Linear volumetric infusion pump ST-1000, Samtronic, São Paulo, SP, Brazil).

Cardiorespiratory variables were recorded immediately before anesthetic induction and at 15, 30, 45, and 60 minutes after the start of continuous propofol infusion (T15, T30, T45, and T60, respectively). Ruminal motility was evaluated before food withholding, which was defined as the beginning of fasting (BF), and at the end of the fasting period (EF). During anesthetic maintenance, ruminal motility was assessed at the same time points used for the remaining variables. During anesthetic recovery, ruminal motility was evaluated every 10 minutes until contractions returned to values equivalent to those observed at BF.

The following variables were evaluated using a multiparameter monitor equipped with invasive blood-pressure and gas-analyzer modules (Multiparameter monitor DX-2020 equipped with invasive blood-pressure and gas-analyzer modules, Dixtal, Manaus, AM, Brazil). Heart rate (HR) was recorded in beats per minute (bpm) using an electrocardiograph adjusted to lead II. SAP, DAP, and MAP were measured invasively (mmHg) using a multiparameter monitor, with the transducer connected to the catheter previously placed in the left auricular artery and positioned at heart level for calibration. Oxyhemoglobin saturation (SpO_2_) was obtained by direct reading from a multiparameter monitor, with the sensor positioned in body regions presenting adequate tissue transparency. End-tidal carbon dioxide concentration (ETCO_2_) and respiratory rate (RR) were measured using a monitor equipped with a sidestream gas analyzer. At BM, the sampling line was positioned near the nostrils; during anesthesia, it was connected to the distal portion of the endotracheal tube.

Ruminal motility was assessed by auscultation using a stethoscope during a 2-min period, always performed by the same evaluator. During anesthetic recovery, ruminal auscultation was performed every 10 min until ruminal activity returned to values equivalent to those observed at BF. This interval was defined as the ruminal normalization time (RNT). Ruminal contractions were converted into a numerical scoring system as follows: complete movement, 1.0; incomplete movement, 0.5; absence of movement, 0.

Data were analyzed using repeated-measures analysis of variance with the MIXED procedure of SAS (Statistical Analysis System), followed by multiple comparisons of means using LSMEANS (Least Squares Means) adjusted by Tukey’s test at a significance level of 5%. Data normality was assessed using the Shapiro-Wilk test. Variables that did not present normal distribution were analyzed using Friedman’s test for comparisons among time points and using Wilcoxon’s test for comparisons between groups. All statistical analyses were performed using SAS 9.4 software.

All experimental procedures were conducted according to the institutional guidelines of São Paulo State University (UNESP). The study was reviewed and approved by the Ethics Committee on Animal Use of São Paulo State University (CEUA/FOA-UNESP), Araçatuba, Brazil (Process No. FOA-1075-2013).

## Results

HR did not differ between groups (Table 1). However, in both groups, HR values at BM were lower than those recorded during the anesthetic period. For arterial pressures, SAP, DAP, and MAP were lower in the FG than in the PG at T45 and T60. Within-group analysis showed that SAP values at BM and T60 were higher than those observed at intermediate time points in both groups. Similarly, DAP and MAP values were higher at T60 in the PG; in the FG, BM values were higher than those recorded during anesthetic maintenance.

**Table 1:** Mean ± standard deviation of heart rate (HR), systolic arterial pressure (SAP), mean arterial pressure (MAP), diastolic arterial pressure (DAP), oxyhemoglobin saturation (SpO_2_), end-tidal carbon dioxide concentration (ETCO_2_), and respiratory rate (RR) in Holstein calves anesthetized with propofol (PG) or propofol associated with fentanyl (FG) during total intravenous anesthesia.

**Variable**	**Group**	**BM**	**T15**	**T30**	**T45**	**T60**
**HR**	PG	74 ± 24 _Ab_	104 ± 20 _Ba_	98 ± 15 _Aab_	106 ± 12 _Aa_	110 ± 10 _Aa_
	FG	72 ± 13 _Ab_	122 ± 33 _Aa_	113 ± 28 _Aa_	112 ± 23 _Aa_	116 ± 23 _Aa_
**SAP**	PG	161 ± 20 _Aa_	129 ± 16 _Ab_	130 ± 13 _Ab_	147 ± 8 _Aab_	152 ± 13 _Aa_
	FG	151 ± 20 _Aa_	124 ± 17 _Ab_	120 ± 18 _Ab_	124 ± 19 _Bb_	136 ± 19 _Bab_
**MAP**	PG	132 ± 18 _Aa_	104 ± 22 _Ab_	104 ± 14 _Ab_	123 ± 10 _Aab_	134 ± 8 _Aa_
	FG	124 ± 14 _Aa_	98 ± 20 _Ab_	93 ± 21 _Ab_	97 ± 20 _Bb_	110 ± 21 _Bab_
**DAP**	PG	106 ± 21 _Aab_	85 ± 24 _Ab_	84 ± 15 _Ab_	105 ± 10 _Aab_	116 ± 10 _Aa_
	FG	96 ± 14 _Aa_	80 ± 20 _Aab_	71 ± 22 _Ab_	77 ± 19 _Bab_	92 ± 24 _Bab_
**SpO_2_**	PG	96 ± 2 _Aa_	87 ± 4 _Ac_	91 ± 4 _Ab_	91 ± 4 _Ab_	93 ± 3 _Aab_
	FG	95 ± 4 _Aa_	84 ± 6 _Ab_	84 ± 10 _Ab_	83 ± 11 _Ab_	85 ± 13 _Ab_
**ETCO_2_**	PG	17 ± 5 _Ab_	54 ± 4 _Aa_	56 ± 4 _Aa_	55 ± 5 _Aa_	54 ± 5 _Aa_
	FG	19 ± 7 _Ab_	55 ± 4 _Aa_	55 ± 5 _Aa_	56 ± 6 _Aa_	53 ± 8 _Aa_
**RR**	PG	42 ± 12 _Aa_	17 ± 2 _Ab_	18 ± 3 _Ab_	19 ± 2 _Ab_	20 ± 3 _Ab_
	FG	38 ± 18 _Aa_	20 ± 6 _Ab_	18 ± 1 _Ab_	18 ± 1 _Ab_	18 ± 1 _Ab_

Note. BM = baseline measurement; T15, T30, T45, and T60 = 15, 30, 45, and 60 min after the start of continuous rate infusion, respectively. Data are presented as mean ± SD. Means followed by different uppercase letters differ between groups within the same time point, whereas means followed by different lowercase letters differ among time points within the same group according to Tukey’s test (P < 0.05).

Furthermore, SpO_2_, ETCO_2_, and RR did not differ between groups. In the PG, SpO_2_ at T15 differed from that at the remaining time points; however, the corresponding BM values differed from those observed at T30 and T45 (Table 1). In the FG, BM values were higher than those recorded during anesthesia.

For ETCO_2_, BM values were lower than those observed during anesthetic maintenance in both groups. Likewise, RR values at BM were significantly higher than those recorded during anesthesia in both groups. Immediately before anesthetic induction, RR values exceeded the physiological range of 10-30 breaths/min (Feitosa, 2014), which was most likely as a consequence of handling-related stress.

The complete suppression of ruminal contractions was observed during anesthetic maintenance (T15-T60), and no contractions were detected by auscultation in either group during this period (Table 2; Figure 2). Ruminal motility scores differed between groups only at T80. Within the PG, ruminal motility scores at T90, T100, and T110 were higher than those observed during anesthetic maintenance (T15-T60). Similarly, in the FG, ruminal motility scores at T15-T60 differed from those observed at T100 and T110; scores at T90 did not differ from those at any other time point.

**Table 2:** Ruminal motility scores in Holstein calves anesthetized with propofol (PG) or propofol-fentanyl (FG) during total intravenous anesthesia.

**Time Point**	**PG**		**FG**		**P** 1
**BF**	1 (0-2)	_Aab_	1.5 (0-3)	_Aab_	0.69
**EF**	0.5 (0-2)	_Aab_	0.5 (0-1.5)	_Aab_	0.38
**BM**	0.5 (0-2.5)	_Aab_	0.25 (0-2)	_Aab_	0.78
**T15**	0 (0-0)	_Ab_	0 (0-0)	_Ab_	-
**T30**	0 (0-0)	_Ab_	0 (0-0)	_Ab_	-
**T45**	0 (0-0)	_Ab_	0 (0-0)	_Ab_	-
**T60**	0 (0-0)	_Ab_	0 (0-0)	_Ab_	-
**T70**	1 (0-2)	_Aab_	0 (0-1)	_Aab_	0.13
**T80**	1.25 (0-2.5)	_Aab_	0.25 (0-2)	_Bab_	0.03
**T90**	1.5 (0-3)	_Aa_	0.77 (0-3)	_Aab_	0.42
**T100**	1.5 (0.5-2)	_Aa_	1 (0.5-3)	_Aa_	0.97
**T110**	1.5 (0.5-2)	_Aa_	1.5 (0.5-3)	_Aa_	1.00
**P** 2	< .0001		< .0001		

Note. BF = beginning of fasting; EF = end of fasting; BM = baseline measurement; T15, T30, T45, and T60 = 15, 30, 45, and 60 min after the start of continuous rate infusion, respectively; T70, T80, T90, T100, and T110 = 10, 20, 30, 40, and 50 min after discontinuation of the anesthetic infusion, respectively.

1 Wilcoxon test.

2 Friedman test. Data are presented as median (minimum-maximum). Different uppercase letters indicate differences between groups within the same time point, whereas different lowercase letters indicate differences among time points within the same group (P < 0.05).

**Figure 2:**
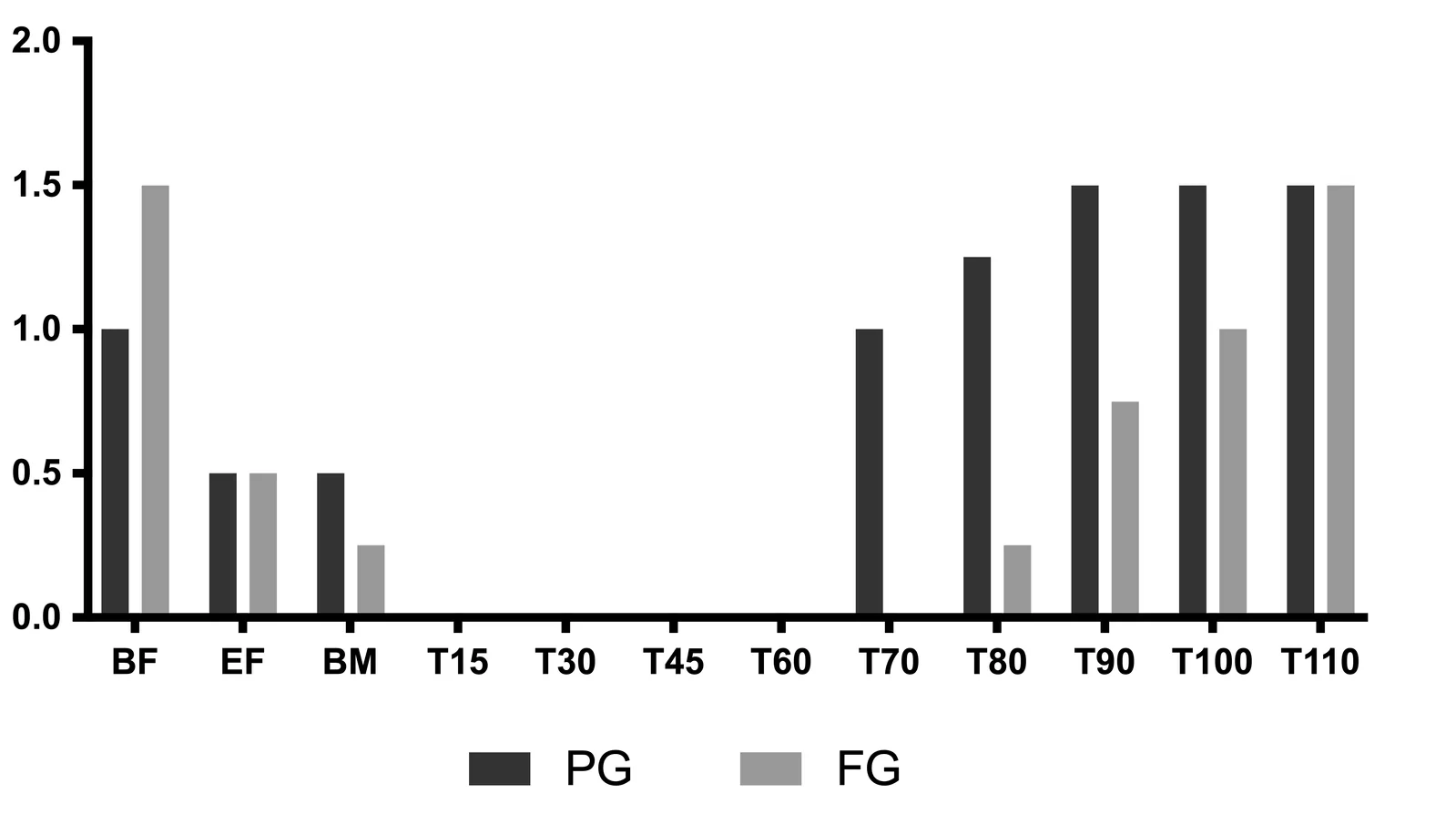
Median ruminal motility scores in Holstein calves anesthetized with propofol (PG) or propofol-fentanyl (FG) during total intravenous anesthesia.

Note. BF = beginning of fasting; EF = end of fasting; BM = baseline measurement; T15, T30, T45, and T60 = 15, 30, 45, and 60 min after the start of continuous rate infusion, respectively; T70, T80, T90, T100, and T110 = 10, 20, 30, 40, and 50 min after discontinuation of the anesthetic infusion, respectively.

The comparison of BF and EF revealed a reduction in ruminal contractions in both groups. Furthermore, in the FG, a partial reduction was observed between EF and BM.

The median (minimum/maximum) RNT was 35 (10/50) minutes in the PG and 45 (20/120) minutes in the FG (P = 0.19). Although no significant difference was detected between groups, the FG showed a wider range of recovery times, mainly because one calf required approximately 120 min after the end of infusion to recover ruminal motility equivalent to that observed at BF. Ruminal contractions began to return approximately 10 min after the end of infusion in the PG and 20 min after the end of infusion in the FG. No clinical complications were observed during the recovery period.

## Discussion

In the present study, no reduction in HR was observed following propofol administration, differing from previous reports describing bradycardia after propofol induction (Manica, 1997). The increase in HR observed herein may be related to a compensatory reflex response to reductions in arterial pressure observed during anesthesia. Fentanyl may promote a reduction in HR (Bovill et al., 1984); however, this effect was not observed in the FG, wherein a relative increase in HR was recorded. Similarly, anesthetic induction with propofol did not significantly affect HR in agoutis (*Dasyprocta sp.)* (Leite et al., 2008).

In both groups, SAP, DAP, and MAP decreased during the anesthetic period. However, these variables were lower in the FG than in the PG at T45 and T60. These findings may be attributed to the dose-dependent vasodilatory effects of propofol, which reduce systemic vascular resistance and thereby arterial pressure, in agreement with results reported in cats (Selmi et al., 2005). The lower arterial-pressure values observed in the FG may also reflect the combined cardiovascular effects of propofol and fentanyl. Nevertheless, because the present study did not include a group receiving fentanyl alone, the independent contribution of the opioid to these changes could not be determined

SpO_2_ decreased during anesthesia in both groups, in parallel with the reduction in RR, consistent with the respiratory depressant effects of propofol (Massone, 2011). Significant respiratory depression has been reported in calves receiving continuous infusions of propofol alone or in combination with fentanyl, while breathing room air (Deschk et al., 2017). Although no significant difference in SpO_2_ was detected between groups in the present study, mean values in the FG were 83-85% during anesthesia, indicating clinically relevant oxyhemoglobin desaturation. These findings support the provision of supplemental oxygen and close respiratory monitoring when these protocols are used in spontaneously breathing calves.

In this study, an increase in ETCO_2_, together with a reduction in RR, indicated marked hypoventilation and carbon dioxide retention during anesthesia. Similar respiratory changes were reported by Deschk et al. (2017) in calves anesthetized with continuous infusions of propofol with or without fentanyl. Although this pattern is compatible with the development of respiratory acidosis, this condition could not be confirmed in the present study because arterial pH and PaCO_2_ were not evaluated. Despite these respiratory alterations, hemodynamic variables were comparatively preserved, and no clinically detectable cardiovascular complications were observed. Nevertheless, the elevated ETCO_2_ and reduced SpO_2_ values emphasize the importance of oxygen supplementation, close respiratory monitoring, and ventilatory-support availability.

Ruminal hypomotility was evident during continuous drug infusion in both groups, with the complete suppression of ruminal contractions during T15, T30, T45, and T60. This finding was expected, owing to depressant effects of anesthetic agents on gastrointestinal motor activity (Feitosa, 2014). Complete suppression occurred with both protocols and was reversible after infusion discontinuation. Furthermore, the absence of a significant difference in RNT between groups indicates that the addition of fentanyl did not exacerbate or prolong ruminal-motility suppression at the group level.

Consistent with pharmacological characteristics of the employed drugs, ruminal contractions began to return approximately 10 min after the end of infusion in the PG and approximately 20 min after the end of infusion in the FG. Although the median RNT did not differ significantly between groups (P = 0.19), the wider range observed in the FG (20-120 min) than in the PG (10-50 min) indicates considerable individual variability. This wider range was mainly influenced by one calf requiring 120 min to recover ruminal motility equivalent to that recorded at BF.

The delayed recovery observed in this animal may have resulted from individual differences in propofol or fentanyl pharmacokinetics and metabolism, sensitivity to anesthetic or opioid effects, baseline ruminal activity, or responses to food withholding and handling-related stress. Individual variability in fentanyl disposition has previously been reported in Holstein calves (Smith et al., 2018), suggesting that pharmacokinetic differences contributed to the recovery time observed in this animal. However, because plasma drug concentrations and metabolic variables were not evaluated in the present study, the precise cause of the prolonged recovery could not be determined. Furthermore, because this finding occurred in only one calf and no significant differences in RNT were detected between groups, the delayed recovery cannot be conclusively attributed to fentanyl addition. Further studies involving larger samples and pharmacokinetic monitoring are warranted to determine the mechanisms and clinical relevance of this individual variability.

One limitation of this study is its small sample size, possibly reducing the study’s ability to detect subtle differences between anesthetic protocols. However, the crossover design of this study minimized interindividual variability by allowing each calf to serve as its own control, strengthening the comparison between anesthetic protocols.

From a clinical perspective, the initial return of ruminal contractions approximately 20 min after discontinuation of the anesthetic infusion in the FG was unlikely to be clinically significant in healthy calves subjected to 60 min of anesthesia, because no cases of bloat or other clinically detectable gastrointestinal complications were observed. However, no studies have established the maximum duration of ruminal inactivity required for secondary bloat development. Moreover, one calf required 120 min to recover its baseline ruminal motility. Therefore, the findings of the present study should not be extrapolated to longer anesthetic procedures or to animals with pre-existing gastrointestinal disorders, and ruminal motility should be carefully monitored during recovery in these circumstances.

## Conclusion

The continuous rate infusion of propofol, with or without fentanyl, completely suppressed ruminal contractions during anesthesia in Holstein calves maintained under spontaneous ventilation. Ruminal contractions began to return approximately 10 min after the end of infusion in the PG and approximately 20 min in the FG. Therefore, although both anesthetic protocols markedly reduced ruminal motility during anesthesia, fentanyl addition was not associated with a statistically significant prolongation of ruminal-motility recovery. Nevertheless, the wider recovery range observed in the FG, including one calf that required 120 min to recover its baseline ruminal motility, suggests individual variability that should be investigated in future studies involving larger samples. Regarding cardiorespiratory variables, both protocols promoted hypoventilation and respiratory acidosis while maintaining HR, SAP, DAP, MAP, and SpO_2_ within clinically acceptable limits throughout the experimental period.
